# Serum Neutrophil Gelatinase-Associated Lipocalin, Total Oxidant Status, and Total Antioxidant Status in Preterm Infants Following Clinical Chorioamnionitis or Preterm Premature Rupture of Membranes: A Multicenter Prospective Cohort Study

**DOI:** 10.3390/children13081044

**Published:** 2026-08-05

**Authors:** Şenol Bozdağ, Sabahattin Ertuğrul, Mustafa Törehan Aslan, Mustafa Aydın, İbrahim Kaplan, Sibel Tanrıverdi Yılmaz

**Affiliations:** 1 Division of Neonatology, Department of Pediatrics, Faculty of Medicine, İstanbul Okan University, 34959 İstanbul, Türkiye; 2 Division of Neonatology, Department of Pediatrics, Faculty of Medicine, Dicle University, 21280 Diyarbakır, Türkiye; sertugrul68@dicle.edu.tr (S.E.); sibel.yilmaz@dicle.edu.tr (S.T.Y.); 3 Division of Neonatology, Department of Pediatrics, School of Medicine, Koç University, 34450 İstanbul, Türkiye; taslan@ku.edu.tr; 4 Division of Neonatology, Department of Pediatrics, Faculty of Medicine, Fırat University, 23119 Elazığ, Türkiye; mustafa.aydin1@sbu.edu.tr; 5 Department of Medical Biochemistry, Faculty of Medicine, Dicle University, 21280 Diyarbakır, Türkiye; ibrahim.kaplan@dicle.edu.tr

**Keywords:** preterm infant, clinical chorioamnionitis, preterm premature rupture of membranes, neutrophil gelatinase-associated lipocalin, oxidative stress, total oxidant status, total antioxidant status, oxidative stress index, necrotizing enterocolitis

## Abstract

**Highlights:**

**What are the main findings?**

- Serum neutrophil gelatinase-associated lipocalin (NGAL) was lowest in preterm infants exposed to clinical chorioamnionitis and significantly lower than in infants exposed to preterm premature rupture of membranes, whereas total oxidant status, total antioxidant status, and the oxidative stress index did not differ by exposure type.
- Serum NGAL separated clinical chorioamnionitis from preterm premature rupture of membranes only modestly (area under the curve, 0.689) and no correlation with conventional inflammatory markers survived correction for multiple comparisons; the strongest single association was with the neutrophil count (ρ = 0.29).

**What are the implications of the main findings?**

- A single early postnatal measurement of serum NGAL or of global oxidant/antioxidant status is unlikely to reliably classify the type of intrauterine inflammatory exposure in extremely preterm infants.
- The lower NGAL in clinical chorioamnionitis-exposed infants is an exploratory, hypothesis-generating observation that requires confirmation in larger studies with serial sampling before any clinical application.

**Abstract:**

**Background/Objectives:** It is unclear whether the type of intrauterine inflammatory exposure influences global oxidant/antioxidant status or neutrophil gelatinase-associated lipocalin (NGAL) in preterm infants. **Methods:** Ninety-four infants born before 32 weeks of gestation were enrolled prospectively at three centers and grouped as clinical chorioamnionitis (*n* = 31), preterm premature rupture of membranes (PPROM; *n* = 33), or controls (*n* = 30) delivered preterm chiefly for preeclampsia, growth restriction, or placental insufficiency. Groups were not matched, but gestational age and birth weight were comparable. Serum total oxidant status (TOS), total antioxidant status (TAS), and NGAL were measured at the 1st postnatal hour. **Results:** TAS, TOS, and the oxidative stress index did not differ among groups (*p* = 0.966, 0.525, and 0.729). NGAL differed (*p* = 0.031) and was lowest in the clinical chorioamnionitis group, confined to the clinical chorioamnionitis-versus-PPROM contrast (adjusted *p* = 0.029) and persisting after multivariable adjustment. After correction for multiple testing, no biomarker correlated with C-reactive protein, blood counts, or procalcitonin at the 1st or 24th postnatal hour, or with the first-hour Töllner sepsis score; the strongest single association was NGAL with the first-hour neutrophil count (ρ = 0.29). Necrotizing enterocolitis was more frequent in controls (*p* = 0.003); mortality (20.2%) did not differ among groups. **Conclusions:** A single early measurement of these biomarkers did not reliably classify the type of intrauterine inflammatory exposure; discrimination was modest (area under the curve, 0.689), so serum NGAL has no current clinical applicability. These hypothesis-generating findings require confirmation in adequately powered studies with serial sampling.

## 1. Introduction

Preterm birth remains a leading cause of neonatal morbidity and mortality worldwide, and preterm infants are particularly vulnerable to oxidative stress owing to immature antioxidant defenses, high oxygen consumption, and the abrupt transition from the relatively hypoxic intrauterine environment to extrauterine life. The resulting imbalance between oxidant production and antioxidant capacity has been implicated in several characteristic complications of prematurity, including bronchopulmonary dysplasia, necrotizing enterocolitis, retinopathy of prematurity, periventricular leukomalacia, and intraventricular hemorrhage [1]. Because many individual oxidant and antioxidant species act simultaneously, the total oxidant status (TOS) and total antioxidant status (TAS) have been introduced as integrated measures of the overall oxidative balance, each of which can be quantified by an automated colorimetric method [2,3].

Intrauterine infection and inflammation are major contributors to spontaneous preterm birth. Chorioamnionitis—inflammation of the fetal membranes, amniotic fluid, and/or decidua—is recognized as a leading cause of very preterm birth and contributes substantially to neonatal morbidity and mortality [4]. Exposure to chorioamnionitis and the accompanying fetal inflammatory response has been associated with an increased risk of preterm labor and early-onset neonatal sepsis [5]. Although histologic examination of the placenta is the diagnostic gold standard, in clinical practice the diagnosis frequently relies on maternal clinical criteria, namely fever accompanied by maternal or fetal tachycardia, uterine tenderness, malodorous amniotic fluid, or maternal leukocytosis [6,7].

Preterm premature rupture of membranes (PPROM), defined as rupture of the fetal membranes before 37 weeks of gestation and prior to the onset of labor, accounts for a substantial proportion of preterm births and is frequently associated with ascending intra-amniotic inflammation and infection [8]. Chorioamnionitis and PPROM therefore represent overlapping yet distinct intrauterine inflammatory exposures that may differentially influence the fetal and neonatal redox and inflammatory milieu.

Neutrophil gelatinase-associated lipocalin (NGAL), a 25 kDa member of the lipocalin family originally isolated from human neutrophils [9], is released by activated neutrophils and epithelial cells. Beyond its established role as an early biomarker of acute kidney injury, NGAL exerts bacteriostatic and growth- and differentiation-modulating actions and is markedly up-regulated during infection and systemic inflammation [10]. In critically ill adults, circulating NGAL increases with the severity of infection and is associated with outcome, albeit with modest discriminative performance [11]. In neonates, serum NGAL has been investigated as an early indicator of sepsis, reflecting neutrophil activation following an infectious stimulus [12], and urinary NGAL has likewise been evaluated as a sepsis marker in preterm infants [13]. Evidence linking NGAL specifically to intrauterine inflammation, however, derives largely from the maternal compartment: in women with preterm premature rupture of membranes, amniotic fluid NGAL is up-regulated in the presence of microbial invasion of the amniotic cavity and of histologically confirmed chorioamnionitis [14]. Whether this intra-amniotic signal is mirrored in the circulation of the exposed neonate, and whether it differs between infants exposed to clinical chorioamnionitis and those exposed to PPROM, has not been established.

Despite the central roles of oxidative stress and intrauterine inflammation in the pathophysiology of prematurity, few studies have simultaneously assessed global oxidant/antioxidant status and NGAL in preterm infants according to the specific type of intrauterine inflammatory exposure. Earlier neonatal work assessed umbilical cord blood oxidative status in infants exposed to PPROM and to the fetal inflammatory response syndrome [15], and methodological reviews have emphasized how strongly oxidative stress biomarker results depend on the biological matrix sampled, the timing of sampling, and the assay selected [16]. To the best of our knowledge, however, no previous study has simultaneously compared serum NGAL, TOS, TAS, and the derived oxidative stress index among preterm infants exposed to clinical chorioamnionitis, to PPROM, and to neither exposure within a single prospective cohort. The specific research question addressed here was therefore whether the type of intrauterine inflammatory exposure is reflected in the early postnatal serum concentrations of these biomarkers in infants born before 32 weeks of gestation. We hypothesized that first-hour serum TOS, TAS, NGAL, and the derived oxidative stress index would differ among the three exposure groups. We therefore conducted a multicenter study to compare serum TOS, TAS, and NGAL among preterm infants born before 32 weeks of gestation to mothers with clinical chorioamnionitis, those born after PPROM, and controls, and to examine the relationship of these biomarkers with clinical and laboratory parameters and with major neonatal outcomes.

## 2. Materials and Methods

### 2.1. Study Design and Setting

This multicenter, prospective, comparative observational study was conducted in the neonatal intensive care units (NICUs) of three tertiary centers in Türkiye—the Divisions of Neonatology, Departments of Pediatrics, Faculties of Medicine of İstanbul Okan University, Dicle University, and Fırat University—between 1 July 2021 and 30 June 2022. The study was approved by the Dicle University Non-interventional Clinical Research Ethics Committee (protocol code 307; 3 June 2021) and was conducted in accordance with the Declaration of Helsinki. The study was coordinated under this single institutional ethics approval. On the basis of that approval, each participating center subsequently obtained its own local institutional and hospital permission and conducted the study according to the approved common protocol and the principles of the Declaration of Helsinki, so that identical eligibility criteria, definitions, sampling procedures, and data-collection forms were applied at all three sites. Written informed consent was obtained from the parents of all participating infants.

### 2.2. Participants and Group Definitions

Infants with a gestational age below 32 weeks who were admitted to the participating NICUs during the study period were eligible. Throughout this report the term clinical chorioamnionitis is used to denote chorioamnionitis diagnosed by maternal clinical criteria; placental histopathological examination was not available for this cohort, and no infant was classified on histological grounds. Gestational age was calculated from the last menstrual period and was confirmed by first-trimester ultrasonography in all included infants. Infants with major congenital anomalies were excluded. Eligible infants were assigned to one of three groups according to maternal status:

*Clinical chorioamnionitis group (n = 31):* infants born to mothers with clinically diagnosed chorioamnionitis, defined as maternal fever (≥38 °C) accompanied by at least two of the following: maternal tachycardia, fetal tachycardia, uterine tenderness, foul-smelling or purulent amniotic fluid, or maternal leukocytosis [6].

*Preterm premature rupture of membranes (PPROM) group (n = 33):* infants born to mothers with rupture of the fetal membranes before 37 weeks of gestation and prior to the onset of labor [8], with a latency of more than 18 h before delivery, in the absence of clinical chorioamnionitis.

*Control group (n = 30):* preterm infants born to mothers without clinical chorioamnionitis or PPROM, who were delivered preterm for other maternal or fetal indications, such as preeclampsia, intrauterine growth restriction, or placental insufficiency.

Because the diagnosis of clinical chorioamnionitis does not require ruptured membranes, whereas the PPROM group was defined by rupture of the membranes with a latency of more than 18 h, the two exposures are not mutually exclusive in origin. The membranes had also ruptured before delivery in 27 of the 31 mothers assigned to the clinical chorioamnionitis group, who were classified on the basis of the clinical diagnosis irrespective of membrane status. The contrast between these two groups should therefore be understood as a comparison between preterm infants exposed to intrauterine inflammation that was clinically apparent and those exposed to ruptured membranes without clinical signs of infection, rather than as a comparison between two wholly separate exposures.

### 2.3. Clinical Variables and Outcome Definitions

Gestational age, birth weight, and mode of delivery were recorded for all infants, together with maternal age, maternal hematological and inflammatory parameters (white blood cell count, lymphocyte count, neutrophil count, hemoglobin, platelet count, and C-reactive protein), and antenatal corticosteroid administration. Neonatal laboratory parameters were obtained at the 1st and 24th postnatal hours. Delivery-room stabilization was standardized across the three participating centers and was performed in full accordance with the American Academy of Pediatrics Neonatal Resuscitation Program (NRP), 8th edition [17]. Resuscitation of infants born before 35 weeks of gestation—a category that included every infant in the present cohort—was initiated with a blended inspired oxygen fraction of 21–30%, and the inspired oxygen fraction was thereafter titrated against preductal oxygen saturation measured by pulse oximetry to meet the minute-specific target saturation ranges specified by the program. Because supplemental oxygen is itself a determinant of postnatal oxidative stress, the uniform application of this protocol at all three sites makes it unlikely that any between-group difference in oxidant or antioxidant status was driven by differing delivery-room oxygen exposure. Bronchopulmonary dysplasia was assessed at 36 weeks’ postmenstrual age and graded according to the mode of respiratory support (no support; nasal cannula ≤2 L/min; nasal cannula >2 L/min or noninvasive positive airway pressure; invasive mechanical ventilation), following the evidence-based definition proposed by Jensen et al. [18]. Necrotizing enterocolitis was diagnosed and staged according to the modified Bell criteria [19]. Patent ductus arteriosus was confirmed by echocardiography. Blood culture positivity, peripheral-smear toxic granulation, and surfactant administration were also recorded. Clinical suspicion of sepsis was quantified at the 1st postnatal hour with the sepsis score of Töllner [20], a composite index in which clinical signs and simple haematological findings are each assigned a weighted number of points, a higher total denoting a greater clinical likelihood of septicaemia. The score was not recalculated at the 24th postnatal hour. In-hospital (pre-discharge) mortality was recorded as a clinical outcome.

### 2.4. Blood Sampling and Biochemical Analyses

Total oxidant status (TOS), total antioxidant status (TAS), and serum neutrophil gelatinase-associated lipocalin (NGAL) were measured in residual serum obtained from blood drawn for routine clinical care, so that no additional blood sampling was required. The biomarker sample was obtained at the 1st postnatal hour. Samples were centrifuged at 3000 rpm for 10 min, and the separated serum was stored at −80 °C until analysis. All assays were performed at the Department of Biochemistry, Dicle University Faculty of Medicine. TAS and TOS were measured using commercial assay kits based on the automated colorimetric methods developed by Erel (Rel Assay Diagnostics, Gaziantep, Türkiye); For TAS, the antioxidative capacity of the sample against hydroxyl radicals generated by a Fenton reaction was measured, and results were expressed as mmol Trolox equivalent/L [3]. For TOS, oxidants in the sample oxidized a ferrous ion–o-dianisidine complex to ferric ion, which formed a colored complex with xylenol orange that was measured spectrophotometrically; the assay was calibrated with hydrogen peroxide, and results were expressed as µmol H_2_O_2_ equivalent/L [2]. In addition, to summarize the oxidant–antioxidant balance as a single integrated measure, the oxidative stress index (OSI) was derived from these two assays and calculated as OSI (arbitrary units) = [TOS (µmol H_2_O_2_ equivalent/L)/TAS (µmol Trolox equivalent/L)] × 100, with TAS converted to µmol/L. Serum NGAL was measured by a commercial sandwich enzyme-linked immunosorbent assay (Sunred, Shanghai, China; catalog no. 201-12-1720) according to the manufacturer’s instructions and expressed as ng/mL.

### 2.5. Statistical Analysis

Statistical analyses were performed using SPSS version 20 (IBM Corp., Armonk, NY, USA), MedCalc version 20.015, and R version 4.6.0 (R Foundation for Statistical Computing, Vienna, Austria). The normality of continuous variables was assessed with the Shapiro–Wilk test; as the data were not normally distributed, non-parametric tests were applied. Continuous variables are presented as median [interquartile range] and categorical variables as counts (percentages). Differences among the three groups were evaluated with the Kruskal–Wallis test, followed by pairwise Mann–Whitney U tests with Bonferroni correction for significant results. Categorical variables were compared using the Pearson chi-square test or Fisher’s exact test where expected cell counts were below five. Correlations between biomarkers and clinical/laboratory variables were assessed using the Spearman rank correlation. Receiver operating characteristic (ROC) curve analysis was used to evaluate the discriminative ability of NGAL. A two-sided *p* < 0.05 was considered statistically significant.

To assess whether the between-group difference in serum NGAL was independent of potential confounders, a multivariable linear regression model was fitted with log-transformed serum NGAL as the dependent variable and study group (control as the reference category), gestational age, birth weight, maternal age, and antenatal corticosteroid exposure as independent variables; the clinical chorioamnionitis-versus-PPROM contrast was estimated from the fitted model. Model assumptions were assessed by inspection of residuals and variance inflation factors, and because the residuals deviated from normality, 95% confidence intervals for the group effects were additionally obtained by group-stratified bootstrap resampling (5000 replicates). The study did not have a single pre-specified primary outcome; the principal research question was the comparison of the early biomarker profile among the three exposure groups. Serum NGAL, TOS, and TAS were regarded as the principal biomarker outcomes, whereas the oxidative stress index was treated as a derived biomarker index calculated from TOS and TAS rather than as an independently measured variable. An a priori sample-size calculation was not performed during the study planning phase; instead, all eligible infants admitted during the predefined enrolment period were consecutively included, and a sensitivity analysis based on the achieved sample size is reported here. For the achieved sample of 94 infants, a sensitivity analysis indicated that, at a two-sided α of 0.05 and 80% power, the study was able to detect an effect size of Cohen’s f = 0.33 for the overall three-group comparison and a standardized between-group difference of Cohen’s d = 0.71 for the clinical chorioamnionitis-versus-PPROM comparison. These thresholds were computed for the parametric analogues of the tests applied—one-way analysis of variance and the two-sample t test—at an uncorrected α, and they are therefore mildly optimistic in two respects: the pairwise comparison was in fact made with a Bonferroni-corrected Mann–Whitney U test, and rank-based tests carry a small loss of efficiency relative to their parametric counterparts. At the Bonferroni-corrected α of 0.017 the detectable standardized difference rises to approximately d = 0.81. This analysis defines the effect sizes that the enrolled cohort was adequately powered to identify and provides additional context for interpreting the findings; conversely, it indicates that smaller—though potentially clinically meaningful—differences could not be excluded, particularly for the low-frequency outcomes of culture-positive sepsis, necrotizing enterocolitis, and in-hospital mortality. Because the effect sizes referred to in the following sentences were estimated from the observed data, these are descriptive statements about the precision the study achieved rather than a post hoc calculation of the power of the tests actually performed, which is already determined by the *p* values reported. To place these thresholds in context, the difference in log-transformed serum NGAL actually observed between the clinical chorioamnionitis and PPROM groups corresponded to a standardized effect size of Cohen’s d = 0.67, for which approximately 35 infants per group would be required for 80% power; the enrolled groups (*n* = 31 and *n* = 33) were therefore close to, although marginally below, the size needed to detect a difference in the magnitude observed. By the same approach, the largest between-group difference in total oxidant status corresponded to d = 0.40 and would have required approximately 98 infants per group, whereas the largest difference in total antioxidant status corresponded to d = 0.07 and would have required more than 3000 infants per group. The null result for total oxidant status may therefore reflect limited power. Although the observed between-group difference in total antioxidant status was very small, the study was not designed as an equivalence study; therefore, smaller potentially meaningful differences cannot be excluded, and the null total antioxidant status finding should be interpreted cautiously. Exploratory associations between the three principal biomarkers (TOS, TAS, and NGAL), together with the derived oxidative stress index, and early (first-hour) clinical and laboratory parameters were assessed with the Spearman rank correlation, and biomarker concentrations were compared between infants with and without culture-positive sepsis, necrotizing enterocolitis, bronchopulmonary dysplasia, and in-hospital mortality using the Mann–Whitney U test. Because these analyses were exploratory and involved multiple comparisons, *p* values were corrected with the Benjamini–Hochberg false-discovery-rate procedure; given the small number of necrotizing enterocolitis events (*n* = 12), no multivariable model of this outcome was fitted. Because C-reactive protein and hematological indices generally require 10–12 h from the onset of an inflammatory stimulus before they become diagnostically informative, this exploratory correlation analysis was extended to the laboratory parameters obtained at the 24th postnatal hour. First-hour serum NGAL, TOS, TAS, and the oxidative stress index were correlated with the 24 h C-reactive protein concentration, white blood cell count, neutrophil count, platelet count, and procalcitonin concentration using the Spearman rank correlation, with the Benjamini–Hochberg false-discovery-rate correction applied across this set of comparisons. Twenty-four-hour hematological data were available for 90 of the 94 infants and 24 h C-reactive protein for 89; the four infants without 24 h hematological values had died before the 24th postnatal hour, and one further infant had no 24 h C-reactive protein measurement. Twenty-four-hour procalcitonin was available in 73 infants because it was not measured routinely at all sites. No repeat biomarker measurement was performed at 24 h, so this analysis relates the first-hour biomarker concentrations to the later conventional laboratory parameters rather than describing the temporal course of the biomarkers themselves.

## 3. Results

### 3.1. Baseline Characteristics

A total of 94 preterm infants born before 32 weeks of gestation were included: 31 in the clinical chorioamnionitis group, 33 in the PPROM group, and 30 controls. Gestational age, birth weight, mode of delivery, and antenatal corticosteroid exposure did not differ significantly among the three groups (Table 1). Maternal age differed across the three groups (Kruskal–Wallis *p* = 0.047), the lowest median being observed in the clinical chorioamnionitis group; because this *p* value lies close to the conventional threshold and six comparisons are reported in Table 1, the difference should be regarded as provisional, and no individual pairwise contrast is claimed. Among the mothers in whom the membranes had ruptured before delivery, the rupture-to-delivery latency was longer in the clinical chorioamnionitis group than in the PPROM group, but the difference was not statistically significant (median 168 h in the 27 mothers with clinical chorioamnionitis and ruptured membranes versus 72 h in the 33 PPROM mothers; *p* = 0.298).

### 3.2. Total Oxidant Status, Total Antioxidant Status, and Neutrophil Gelatinase-Associated Lipocalin

**Figure 1:**
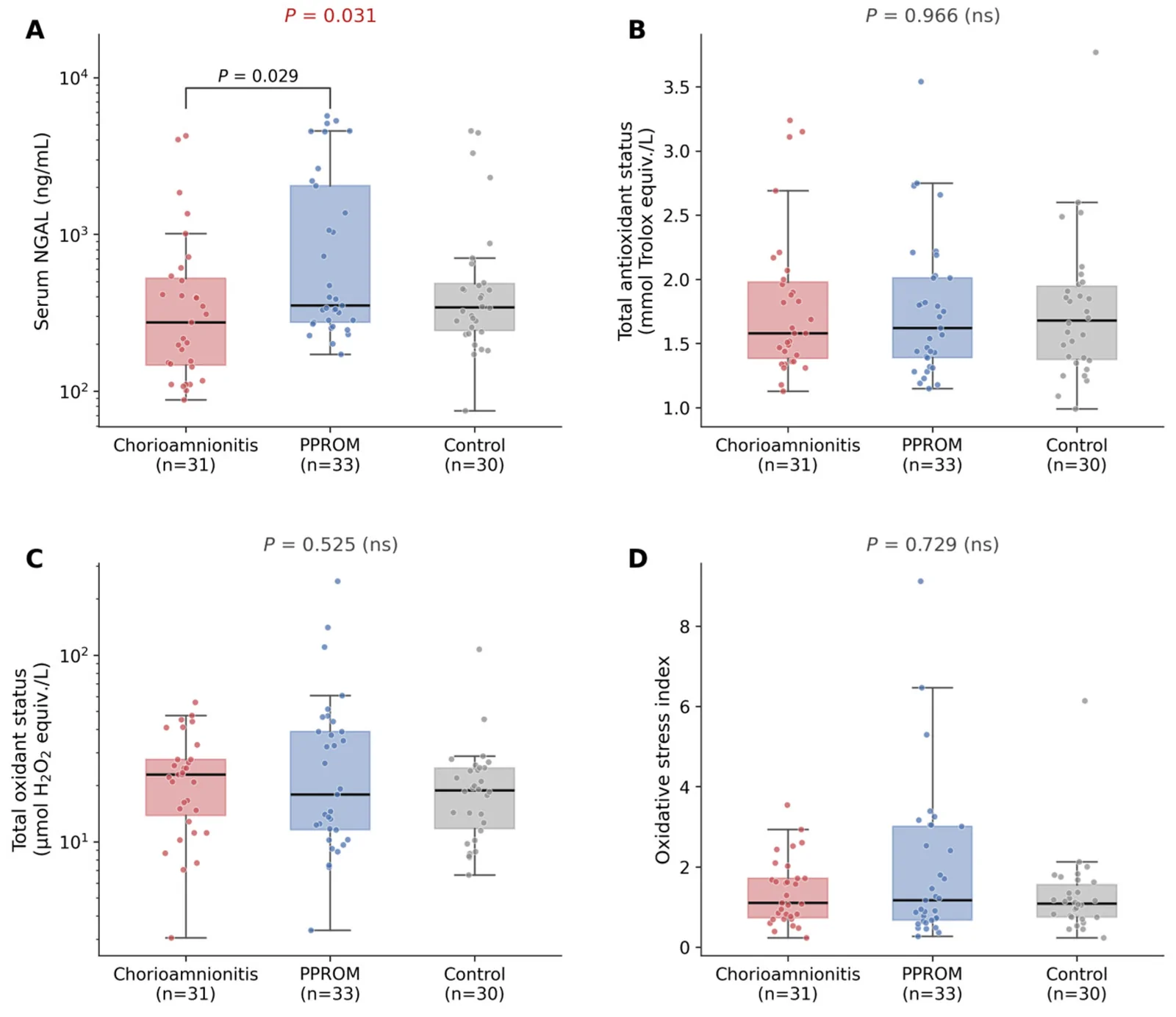
Distribution of serum neutrophil gelatinase-associated lipocalin (**A**), total antioxidant status (**B**), total oxidant status (**C**), and oxidative stress index (**D**) across the three study groups. Boxes denote the median and interquartile range with whiskers extending to 1.5 times the interquartile range, and individual data points are overlaid. The y-axis is logarithmic for neutrophil gelatinase-associated lipocalin and total oxidant status. *p* values are from Kruskal–Wallis tests; the bracket in panel A indicates the Bonferroni-adjusted pairwise comparison between the clinical chorioamnionitis and preterm premature rupture of membranes (PPROM) groups.

**Figure 2:**
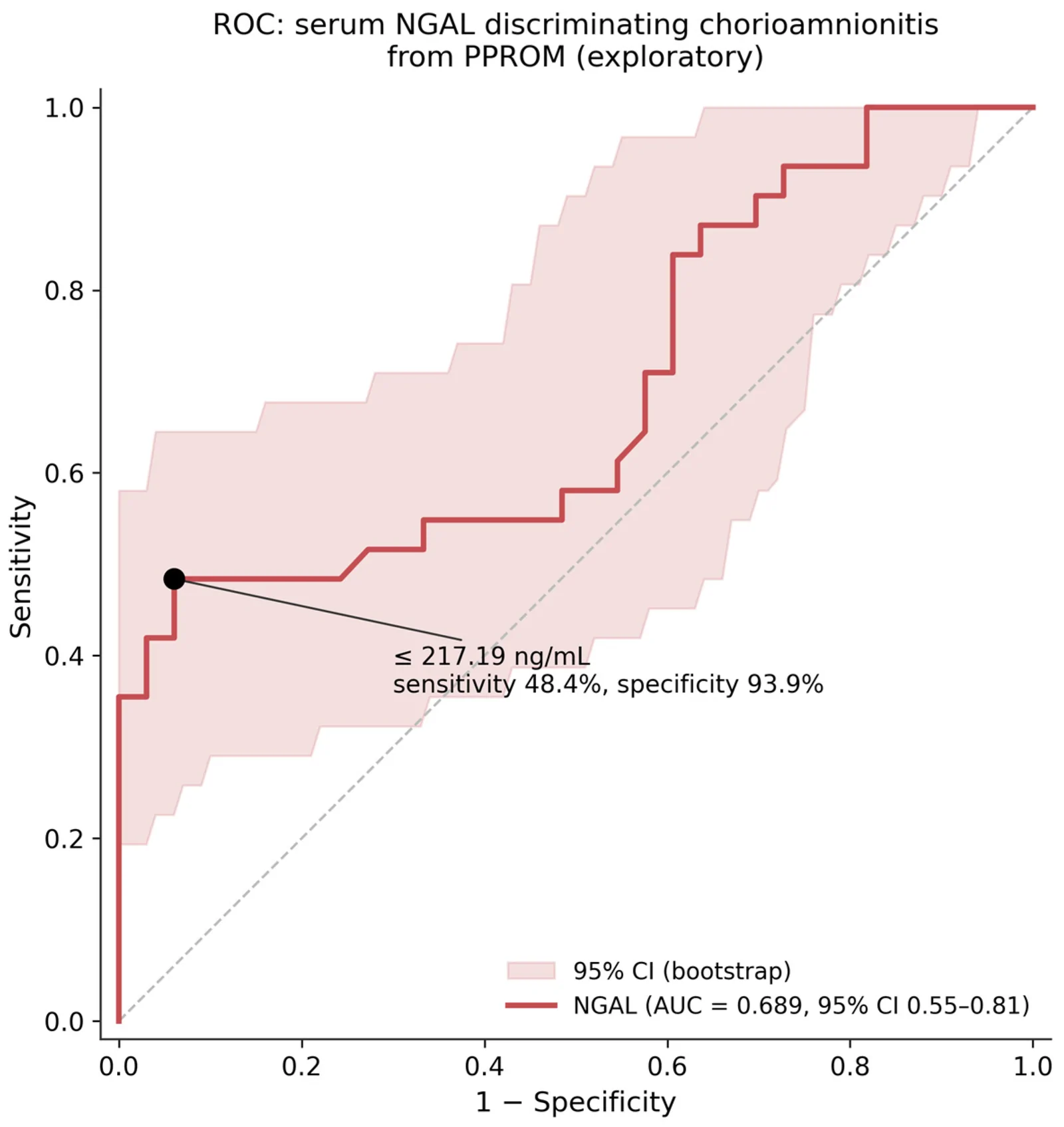
Receiver operating characteristic curve of serum neutrophil gelatinase-associated lipocalin for the discrimination of clinical chorioamnionitis-exposed from preterm premature rupture of membranes (PPROM) -exposed infants (area under the curve, 0.689; 95% confidence interval 0.55–0.81). The shaded band is the bootstrap 95% confidence interval, and the marked point indicates the exploratory optimal cut-off (≤217.19 ng/mL; sensitivity 48.4%, specificity 93.9%).

Total antioxidant status and total oxidant status did not differ significantly among the three groups (Table 2). The oxidative stress index did not differ among the groups either (median 1.11, 1.18, and 1.09 arbitrary units in the clinical chorioamnionitis, PPROM, and control groups, respectively; Kruskal–Wallis *p* = 0.729; Table 2). In contrast, serum neutrophil gelatinase-associated lipocalin (NGAL) differed significantly (*p* = 0.031) and was lowest in the clinical chorioamnionitis group. Pairwise comparison localized this difference to the clinical chorioamnionitis versus PPROM contrast (*p* = 0.010; Bonferroni-adjusted *p* = 0.029), whereas the clinical chorioamnionitis–control and PPROM–control comparisons were not significant (Figure 1). In ROC analysis, serum NGAL discriminated clinical chorioamnionitis-exposed from PPROM-exposed infants with an area under the curve of 0.689; at a cut-off of ≤217.19 ng/mL, sensitivity was 48.4% and specificity was 93.9% (Figure 2). This cut-off was derived from the same sample and is exploratory; it is not intended for clinical use. Serum NGAL showed considerable within-group dispersion that was most pronounced in the PPROM group (interquartile range 275.3–2041.8 ng/mL, versus 146.1–525.6 ng/mL in the clinical chorioamnionitis group and 243.8–486.8 ng/mL in controls), indicating substantial biological heterogeneity within the PPROM-exposed population.

**Figure 3:**
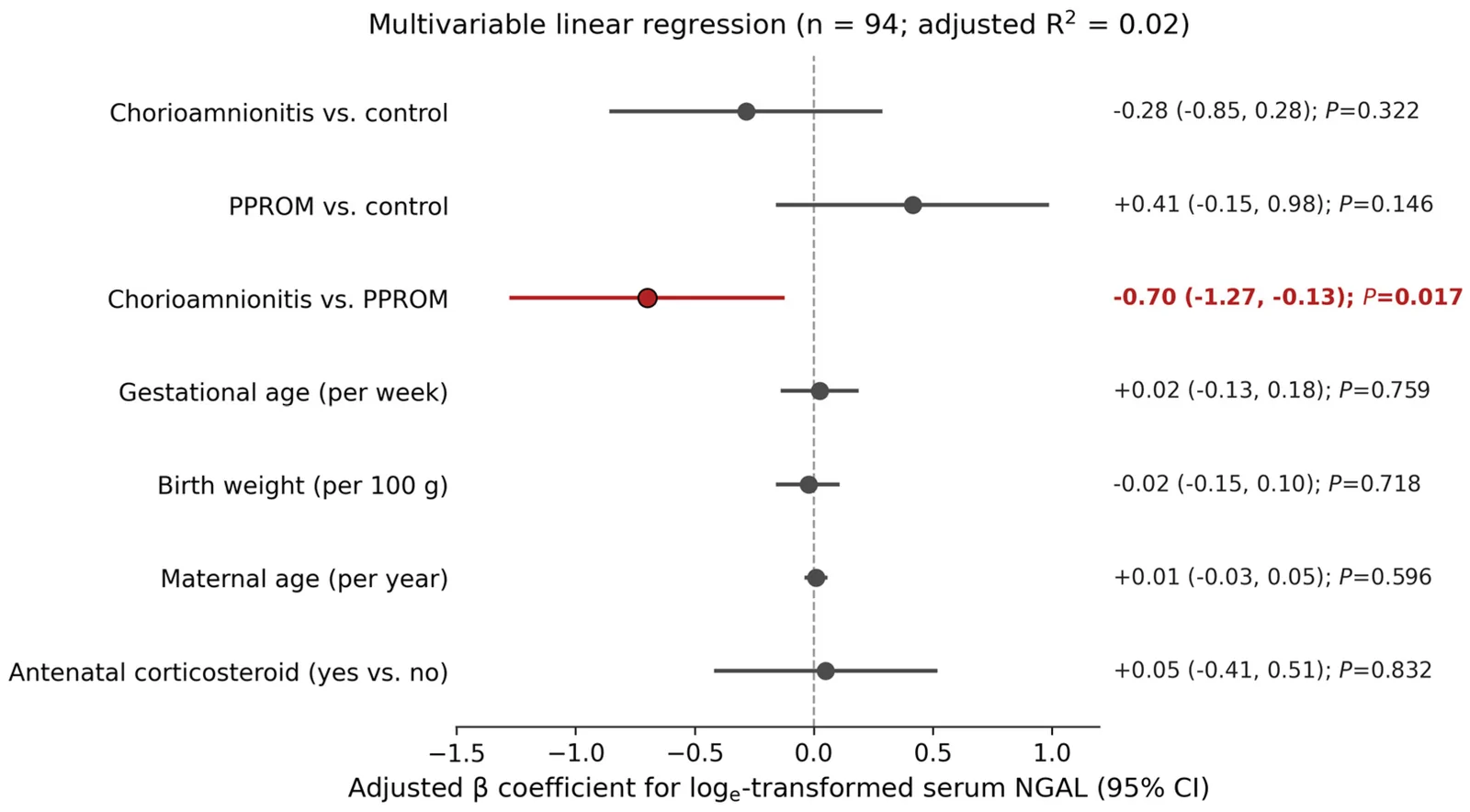
Multivariable linear regression model for the natural-logarithm-transformed serum neutrophil gelatinase-associated lipocalin concentration (*n* = 94). Points and horizontal lines denote the adjusted β coefficients and their 95% confidence intervals; the dashed vertical line marks the null value. The contrast between the clinical chorioamnionitis and preterm premature rupture of membranes (PPROM) groups was the only statistically significant association (highlighted). The model explained little overall variance (adjusted R^2^ = 0.02).

In a multivariable linear regression adjusting for gestational age, birth weight, maternal age, and antenatal corticosteroid exposure, serum NGAL remained significantly lower in the clinical chorioamnionitis group than in the PPROM group (adjusted β for log-NGAL = −0.70; 95% CI, −1.27 to −0.13; bootstrap 95% CI, −1.29 to −0.14; *p* = 0.017), corresponding to an approximately 50% lower adjusted NGAL concentration (e^β^ = 0.50; Table 3 and Figure 3). None of the covariates was independently associated with NGAL; in particular, maternal age was not a significant predictor (β = 0.010; *p* = 0.60), and the model explained little of the overall variance (adjusted R^2^ = 0.02). The persistence of the clinical chorioamnionitis–PPROM difference after adjustment indicates that the association was not explained by maternal age in the fitted model.

### 3.3. Neonatal Outcomes and Mortality

Neonatal outcomes are summarized in Table 4. The rates of bronchopulmonary dysplasia, patent ductus arteriosus, surfactant administration, blood culture positivity, and the Töllner sepsis score did not differ significantly among the groups. Necrotizing enterocolitis was significantly more frequent in the control group than in the clinical chorioamnionitis and PPROM groups (30.0% vs. 3.2% and 6.1%; *p* = 0.003), and peripheral-smear toxic granulation was most frequent in the PPROM group, although the overall comparison was only marginally significant (*p* = 0.049) and no individual pairwise contrast is claimed. Nine of the twelve necrotizing enterocolitis cases occurred in the control group, in which preterm delivery had been indicated by preeclampsia, intrauterine growth restriction, or placental insufficiency. Infants who developed necrotizing enterocolitis were significantly more immature than those who did not (median gestational age 25.0 [24.0–26.5] versus 27.5 [25.3–28.0] weeks; *p* = 0.033), and tended to have a lower birth weight (855 [710–988] versus 1025 [830–1223] g; *p* = 0.068). In-hospital mortality was 20.2% (19/94) overall and was concentrated in the most immature infants—32.0% (16/50) among infants born before 28 weeks versus 6.8% (3/44) among those born at 28–31 weeks (Table 5). Mortality did not differ significantly among the clinical chorioamnionitis, PPROM, and control groups (5/31, 16.1%; 8/33, 24.2%; and 6/30, 20.0%, respectively; *p* = 0.721; Table 4).

### 3.4. Correlations

Correlations between the biomarkers and clinical/laboratory variables were generally weak and mostly non-significant (Table 6). Total antioxidant status and total oxidant status were weakly positively correlated (ρ = 0.25, *p* = 0.017), whereas neutrophil gelatinase-associated lipocalin correlated with neither oxidative marker. Serum NGAL did not correlate with C-reactive protein, white blood cell count, procalcitonin, or the Töllner sepsis score (all *p* ≥ 0.16 before correction and all false-discovery-rate-adjusted q > 0.05). The single exception among the conventional inflammatory indices was the absolute neutrophil count, with which serum NGAL showed a weak positive correlation (ρ = 0.29, *p* = 0.005); this was the smallest uncorrected *p* value in the table, but it did not survive false-discovery-rate correction (q = 0.17). Total oxidant status showed a weak positive correlation with birth weight (ρ = 0.28, *p* = 0.007).

When the three principal biomarkers (TOS, TAS, and NGAL) and the derived oxidative stress index were additionally correlated with first-hour hematological (white blood cell, neutrophil, and platelet counts), biochemical (lactate dehydrogenase, lactate, albumin, and pH), and acute-phase (C-reactive protein and procalcitonin) parameters, as well as the Töllner sepsis score, all associations were weak (|ρ| ≤ 0.29, excluding the mathematically constrained correlations among total oxidant status, total antioxidant status, and the derived oxidative stress index) and none remained statistically significant after Benjamini–Hochberg false-discovery-rate correction (Table 6), indicating no robust relationship between systemic redox status or NGAL and early clinical or laboratory parameters. Six of the 48 coefficients reached nominal significance at *p* < 0.05, against 2.4 expected by chance alone, so the presence of a small number of true but weak associations cannot be excluded; none, however, survived correction. The Töllner sepsis score showed a pronounced floor effect in this cohort, the median being 1.0 in all three groups and both quartiles 1.0 in the clinical chorioamnionitis group, so its correlations with the biomarkers are uninformative rather than evidence of independence.

Serum TOS, TAS, OSI, and NGAL also did not differ significantly between infants with and without culture-positive sepsis, necrotizing enterocolitis, bronchopulmonary dysplasia, or in-hospital mortality. Although NGAL tended to be lower in infants with culture-positive sepsis or necrotizing enterocolitis, and total antioxidant status tended to be lower in those who developed bronchopulmonary dysplasia, none of these differences reached significance after false-discovery-rate correction (all adjusted *p* > 0.05; Table 7).

### 3.5. Exploratory Correlations with Laboratory Parameters at the 24th Postnatal Hour

Because C-reactive protein and the hematological indices used to identify neonatal sepsis generally become informative only some 10–12 h after the onset of an inflammatory stimulus, the first-hour biomarkers were additionally correlated with the corresponding laboratory parameters measured at the 24th postnatal hour (Table 8). Twenty-four-hour hematological values were available for 90 of the 94 infants and 24 h C-reactive protein for 89; the four infants without 24 h hematological values had died before the 24th postnatal hour, and one further infant had no 24 h C-reactive protein measurement. All associations were weak (|ρ| ≤ 0.19), and none attained statistical significance either before or after Benjamini–Hochberg correction (all *p* ≥ 0.066; all false-discovery-rate-adjusted q ≥ 0.539). The strongest associations observed were weak positive correlations between serum neutrophil gelatinase-associated lipocalin and the 24 h neutrophil count (ρ = 0.19, *p* = 0.066) and white blood cell count (ρ = 0.17, *p* = 0.108), together with weak inverse correlations between the 24 h C-reactive protein concentration and both total oxidant status (ρ = −0.18, *p* = 0.092) and the oxidative stress index (ρ = −0.17, *p* = 0.108). These results closely parallel those obtained with the first-hour parameters (Table 6) and indicate that the absence of a relationship between the early biomarkers and conventional inflammatory indices was not attributable to the timing of the comparator measurements. No repeat biomarker measurement was performed at 24 h, so this analysis does not describe the temporal course of the biomarkers themselves.

## 4. Discussion

In this multicenter study of preterm infants born before 32 weeks of gestation, serum neutrophil gelatinase-associated lipocalin (NGAL) differed according to the type of intrauterine inflammatory exposure, being lowest in infants born to mothers with clinical chorioamnionitis and significantly lower than in the PPROM group, whereas total oxidant status (TOS) and total antioxidant status (TAS) were comparable across all three groups. NGAL did not correlate with conventional inflammatory markers, and its ability to separate clinical chorioamnionitis- from PPROM-exposed infants was modest. Taken together, these findings suggest that, in this population, serum NGAL did not behave as a straightforward marker of intrauterine infection.

The direction of the NGAL difference is, at first sight, counterintuitive. NGAL is up-regulated by activated neutrophils and epithelial cells during infection and systemic inflammation [9,10], and circulating NGAL rises with the severity of infection in critically ill patients [11] and in neonates with sepsis [12]. One might therefore have expected the highest, rather than the lowest, NGAL concentrations in the clinical chorioamnionitis group. Several considerations may help explain this discrepancy. First, NGAL is not a specific infection marker; as detailed by Romejko et al., it also functions as an iron-trafficking, bacteriostatic, and epithelial growth- and differentiation-modulating protein, and its serum concentration reflects a balance of renal, hepatic, and epithelial sources rather than neutrophil activation alone [10]. Second, the timing of sampling relative to the inflammatory insult is critical: in adults with sepsis, the separation in NGAL concentrations between outcome groups emerged only on the second and seventh days rather than at presentation [11], so a single early postnatal measurement may not capture an exposure that began in utero at a variable and unknown interval before birth. Third, the difference we observed may reflect relatively higher NGAL in the PPROM group rather than a true suppression in clinical chorioamnionitis; the wide interquartile range in the PPROM group is consistent with this interpretation. This possibility receives some support from the maternal compartment, in which amniotic fluid NGAL is up-regulated in women with PPROM who have microbial invasion of the amniotic cavity or histologically confirmed chorioamnionitis [14]. A rising intra-amniotic signal need not translate into a proportionate rise in the neonatal circulation, and the contrast between the two compartments suggests that amniotic fluid and neonatal serum NGAL should not be regarded as interchangeable measures. We emphasize, however, that each of these explanations remains hypothetical. Our study sampled neither the amniotic fluid nor the placenta, included no serial measurements, and therefore provides no direct evidence of transcriptional suppression, of a timing effect, or of a difference in the tissue source of NGAL between the two exposures. The lower concentration observed in the clinical chorioamnionitis group is accordingly best regarded as an unexplained association rather than a demonstrated biological mechanism, and exposure misclassification, variation in the interval between the onset of intrauterine inflammation and delivery, and residual confounding remain equally plausible explanations. A further consideration specific to the design bears directly on this contrast. Because the diagnosis of clinical chorioamnionitis does not require ruptured membranes, whereas the PPROM group was defined by rupture with a latency exceeding 18 h, the two exposures are not disjoint: The membranes had also ruptured before delivery in 27 of the 31 mothers assigned to the clinical chorioamnionitis group. Among these mothers, the median rupture-to-delivery latency was numerically longer than in the PPROM group, although the difference was not statistically significant (168 versus 72 h; *p* = 0.298). The comparison that generated our principal finding is therefore to a considerable extent, a contrast within the population of preterm infants exposed to ruptured membranes—between those whose mothers developed clinical signs of infection and those whose mothers did not—rather than a contrast between two wholly separate exposures. This has two consequences. It narrows the biological distance between the groups, which would tend to attenuate rather than to create a difference. It also makes the timing explanation advanced above concrete rather than merely hypothetical: if serum NGAL rises and subsequently falls after the onset of intra-amniotic inflammation, infants sampled after a longer latency would be expected to have lower first-hour concentrations. We could not test this directly, because the onset of inflammation, as distinct from the rupture of the membranes, is not observable.

The absence, after correction for multiple comparisons, of any correlation between NGAL and C-reactive protein, white blood cell count, procalcitonin, or the Töllner sepsis score suggests that, in our cohort, NGAL was not tracking acute bacterial infection. One partial exception deserves comment, because it is the association that the biology would predict: serum NGAL correlated weakly with the absolute neutrophil count at the 1st postnatal hour (ρ = 0.29, *p* = 0.005), and a weak positive association in the same direction was present with the 24 h neutrophil count (ρ = 0.19, *p* = 0.066). NGAL is a secondary-granule protein of the neutrophil, so a modest relationship with circulating neutrophil mass is to be expected. The finding is nonetheless of uncertain significance, since it survived false-discovery-rate correction in neither analysis (q = 0.17 and q ≥ 0.539) and a correlation of this magnitude accounts for well under a tenth of the variance in serum NGAL. We report it explicitly rather than omit it, because it was the strongest single association we observed and because it suggests that whatever residual signal serum NGAL carries in this population is more likely to reflect neutrophil mass than the type of intrauterine inflammatory exposure. These correlations relate the first-hour biomarker concentrations to clinical and laboratory parameters that were themselves obtained at the 1st postnatal hour. Because C-reactive protein and the hematological indices used to identify sepsis typically become informative only some 10–12 h after the onset of an inflammatory stimulus, a comparison confined to the first hour could in principle underestimate a genuine association. We therefore repeated the analysis against the corresponding parameters measured at the 24th postnatal hour, by which time an early-onset inflammatory response would be expected to be reflected in these markers. The results were essentially unchanged: every correlation remained weak (|ρ| ≤ 0.19) and none reached significance either before or after false-discovery-rate correction (Table 8). The concordance between the first-hour and 24 h analyses makes it unlikely that the absence of an association was an artefact of the timing of the comparator measurements, and it strengthens the conclusion that a single early measurement of serum NGAL or of global redox status does not track the conventional laboratory features of neonatal infection in this population. It should be emphasized that no repeat biomarker measurement was performed at 24 h; this analysis therefore addresses the timing of the comparator parameters and not the temporal course of NGAL, TOS, or TAS themselves. This is consistent with the broader literature: even where NGAL discriminates infected from non-infected neonates, its relationship with established inflammatory markers is inconsistent, and its performance as a stand-alone marker is limited [11,13]. In the present study, receiver operating characteristic analysis yielded an area under the curve of 0.689 for the distinction between clinical chorioamnionitis and PPROM, with high specificity (93.9%) but low sensitivity (48.4%) at the optimal cut-off—performance comparable to the modest discriminative values reported for NGAL in adult sepsis [11]. Such a profile is insufficient for NGAL to serve as a reliable classifier of the type of intrauterine inflammatory exposure on its own. The between-group NGAL difference and the associated cut-off are therefore exploratory and hypothesis-generating and should not be applied in clinical practice. It is also relevant that neonatal NGAL concentrations may be influenced by non-infectious perinatal factors, including asphyxia and its treatment [21], which would add variability that further attenuates its discriminative ability.

Our finding that TOS and TAS did not differ among the groups indicates that the global oxidative balance, as captured by these integrated measures, was not detectably altered by the type of intrauterine inflammatory exposure in this cohort. Although oxidative stress is firmly implicated in the morbidities of prematurity [1], the present data suggest that exposure to clinical chorioamnionitis or PPROM does not, by itself, produce a measurable shift in serum TOS or TAS at birth—possibly because extreme prematurity itself imposes a dominant oxidative burden across all groups, or because these summary measures are insensitive to compartment-specific or transient changes. The weak positive correlation between TOS and birth weight, and between TOS and TAS, should be regarded as exploratory and hypothesis-generating only. Across a broader panel of first-hour hematological, biochemical, and acute-phase parameters, and across major neonatal outcomes, the oxidant, antioxidant, and NGAL measures showed no robust associations after correction for multiple comparisons, suggesting that a single early measurement of systemic redox status does not track concurrent clinical or laboratory severity in this extremely preterm population. Consistent with the unchanged TOS and TAS, the oxidative stress index—an integrated marker of the oxidant–antioxidant balance that is widely applied in preterm infants [22,23]—also did not differ among the groups, reinforcing that the overall redox balance was comparable across the three types of intrauterine inflammatory exposure. These observations may be set against the small number of neonatal studies that have examined global redox status after intrauterine inflammatory exposure in very preterm infants. Özalkaya et al. measured total antioxidant capacity, total oxidant status, and paraoxonase-1 in umbilical cord blood from 51 preterm infants below 35 weeks of gestation, classified according to PPROM and to the fetal inflammatory response syndrome, the latter defined by an umbilical cord interleukin-6 concentration above 11 pg/mL [15]. Several features of that study differ from ours and may account for the divergent findings: cord blood rather than neonatal serum was analysed, the oxidative stress index was expressed as the ratio of antioxidant to oxidant capacity rather than the converse, exposure was defined by a fetal cytokine threshold rather than by maternal clinical criteria, and the gestational-age range extended to 35 weeks, whereas our cohort was confined to infants below 32 weeks with a median gestational age of 26–27 weeks. As Sánchez-Illana et al. have emphasized in their review of oxidative stress biomarkers in the preterm infant, comparisons across such studies are strongly determined by the biological matrix sampled, the interval between the insult and sampling, and the analytical method selected, and integrated indices such as TOS and TAS may lack the sensitivity to detect compartment-specific or short-lived changes [16]. Evidence obtained specifically in infants born before 32 weeks of gestation points in the same direction: in an inception cohort of premature neonates that included 26 infants born before 32 weeks, cord serum antioxidant capacity correlated with gestational age but, once gestational age was accounted for, showed no association with chorioamnionitis, preeclampsia, or the oxygen radical diseases of prematurity [24]. That observation parallels our own finding that maturity, rather than the type of intrauterine inflammatory exposure, dominates the early antioxidant profile of the very preterm infant. A consideration specific to the present cohort is that extreme prematurity may itself impose an oxidative burden of sufficient magnitude to obscure any additional contribution from the type of intrauterine inflammatory exposure.

A particularly noteworthy and counterintuitive observation was the higher frequency of necrotizing enterocolitis in the control group than in the clinical chorioamnionitis or PPROM groups. This contrasts with the national cohort study of Farghaly et al., in which chorioamnionitis was significantly, albeit modestly, associated with an increased incidence of necrotizing enterocolitis (adjusted odds ratio 1.12) [25]. More recent evidence remains mixed: a 2025 multicenter cohort reported that maternal chorioamnionitis was associated with necrotizing enterocolitis in very preterm infants [26], whereas a Bayesian meta-analysis found that the fetal inflammatory response did not add appreciable short-term risk beyond chorioamnionitis itself [27]. Our result should be interpreted with considerable caution: the number of events was small, the comparison was unadjusted, and several established determinants of necrotizing enterocolitis—including feeding practice, postnatal sepsis, and patent ductus arteriosus—were not controlled for. Importantly, the control infants were delivered preterm for indications such as preeclampsia, intrauterine growth restriction, and placental insufficiency, which are themselves recognized risk factors for necrotizing enterocolitis; the higher rate in the control group is therefore most plausibly explained by confounding by delivery indication rather than by the absence of intrauterine inflammation. Consistent with this, infants who developed necrotizing enterocolitis were more immature than those who did not, and oxidant, antioxidant, and NGAL concentrations did not differ by necrotizing enterocolitis status (Table 7). Chance, residual confounding, and the modest sample size are the most plausible explanations, and this finding should be viewed as hypothesis-generating rather than as evidence against the established chorioamnionitis–necrotizing enterocolitis association. The composition of the control group warrants particular emphasis, because it bears directly on the interpretation of our null oxidative-stress findings. The controls were not healthy preterm infants; by design they were infants delivered preterm for maternal or fetal indications, principally preeclampsia, intrauterine growth restriction, and placental insufficiency. Each of these conditions is itself associated with placental oxidative stress, systemic inflammation, and an adverse neonatal risk profile. A comparison group of this composition does not represent an oxidatively unstressed reference population, and its use plausibly narrowed the contrast in TOS, TAS, and the oxidative stress index between exposed and non-exposed infants. The absence of a between-group difference in these measures should therefore be read as the absence of a difference relative to a comparison group that was itself oxidatively stressed, rather than as evidence that intrauterine inflammation leaves global redox balance unaffected. The same consideration applies to the excess of necrotizing enterocolitis in the control group, which is most plausibly explained by confounding by delivery indication and by the greater immaturity of the affected infants. Because the specific delivery indication was not recorded in a form permitting statistical adjustment, we could not control for it, and this residual confounding by indication is a principal limitation of the present analysis. A more informative design would compare each inflammatory exposure both with infants delivered for non-inflammatory indications and with preterm infants free of placental vascular disease.

In-hospital mortality in our cohort was strongly gradient-dependent on gestational age, being far higher among infants born before 28 weeks than at 28–31 weeks, as expected for an extremely preterm population. By contrast, mortality did not differ among the clinical chorioamnionitis, PPROM, and control groups. Gestational age and birth weight did not differ significantly either, which makes gross confounding by maturity less likely; the absence of a significant difference in these baseline variables is not, however, evidence of their equivalence, and with 19 deaths in total the mortality comparison had very little power to detect anything other than a large difference. Maternal age showed a marginal overall difference across the three groups (*p* = 0.047), although no individual pairwise contrast was established; it was therefore retained as a potential confounder in the multivariable analysis [4,5].

This study has several strengths, including its multicenter, prospective design, the simultaneous assessment of oxidant/antioxidant status and NGAL, and the use of standardized methods—the Erel assays for TOS and TAS [2,3] and a Töllner-based sepsis assessment comparable to that used in earlier neonatal NGAL studies [13,28]. Nonetheless, important limitations must be acknowledged. NGAL was the only one of the three biomarkers to show a significant between-group difference, and given that multiple comparisons were performed without global correction beyond the pairwise post hoc analysis, the possibility of a chance finding cannot be excluded. This concern merits explicit statement. The three principal biomarkers and the derived oxidative stress index were compared across the three groups, and serum NGAL was the only one to attain significance, at a *p* value (0.031) close to the conventional threshold; had a formal correction been applied across these four comparisons, the result would not have remained significant. The finding is therefore compatible with a type I error arising from multiple testing. Two observations argue partially against a purely spurious result—the difference persisted after multivariable adjustment and under bootstrap resampling, and it was confined to a single, biologically coherent pairwise contrast—but neither addresses the multiplicity problem directly. Moreover, the adjusted model accounted for only about 2% of the variance in log-transformed NGAL (adjusted R^2^ = 0.02). A model of such low explanatory value indicates that the great majority of between-individual variation in serum NGAL was determined by factors not captured by exposure group, gestational age, birth weight, maternal age, or antenatal corticosteroid exposure. The group difference, although statistically detectable, is consequently of limited mechanistic significance and should not be taken as evidence that the type of intrauterine exposure is an important determinant of circulating NGAL. The receiver operating characteristic cut-off was derived from the same sample without internal or external validation and is therefore likely optimistic. Although the clinical chorioamnionitis–PPROM difference in serum NGAL persisted in a multivariable model adjusting for maternal age and other covariates (Table 3), that model explained little of the variance, and residual confounding cannot be excluded. The single early postnatal measurement cannot capture the temporal dynamics of NGAL, the sample size was modest, the intrauterine inflammatory exposure was defined clinically rather than histologically, which is known to have limited diagnostic accuracy [7], and the observational design precludes causal inference. Beyond these, placental histopathological examination was not routinely performed in the participating centers during the study period, so that no histological data were available for this cohort and the exposure classification rests entirely on maternal clinical criteria. Clinical chorioamnionitis has both limited sensitivity and limited specificity relative to the histological diagnosis; some infants classified as exposed to clinical chorioamnionitis are therefore likely to have had no histological inflammation, while some infants assigned to the PPROM and control groups are likely to have had unrecognized histological chorioamnionitis or funisitis. Misclassification of this kind would be expected to be non-differential with respect to the biomarkers and would therefore tend to attenuate genuine between-group differences towards the null; it may thus have contributed to the absence of differences in TOS, TAS, and the oxidative stress index, and it may equally have distorted the magnitude or even the direction of the NGAL difference we observed. In addition, we did not measure interleukin-6, interleukin-8, or tumour necrosis factor-α; although procalcitonin was available at the 1st and 24th postnatal hours, this limited panel does not permit us to determine whether serum NGAL in this setting reflects inflammation, oxidative injury, tissue damage, or an independent biological pathway. Resolving this question will require the concurrent measurement of a broader cytokine panel. A single first-hour sample likewise cannot describe the trajectory of a dynamic protein, and a design incorporating cord blood together with sampling at 1, 24, and 72 h and on day 7 would be considerably better suited to capturing the peak, the resolution, and any delayed neonatal activation of the NGAL response. These considerations indicate that our findings are hypothesis-generating and require confirmation in larger cohorts with serial biomarker sampling and with systematic placental histopathology combined with amniotic fluid or cord blood cytokine measurement.

## 5. Conclusions

In this multicenter cohort of preterm infants born before 32 weeks of gestation, serum neutrophil gelatinase-associated lipocalin was lowest in those exposed to clinical chorioamnionitis and was significantly lower than in infants born after preterm premature rupture of membranes. This difference persisted after adjustment for maternal age and other covariates (Table 3). By contrast, total oxidant status, total antioxidant status, and the oxidative stress index did not differ according to the type of intrauterine inflammatory exposure. Neutrophil gelatinase-associated lipocalin did not correlate with conventional inflammatory markers, and its ability to discriminate between exposure groups was modest, indicating that it did not behave as a straightforward marker of intrauterine infection in this setting. Discrimination between the clinical chorioamnionitis and PPROM groups was limited (area under the curve, 0.689, with a sensitivity of 48.4% at the exploratory cut-off), which corresponds to poor-to-fair performance. On the basis of these data, a single measurement of serum NGAL in the first postnatal hour has no current clinical applicability for classifying the type of intrauterine inflammatory exposure and should not be used for this purpose outside a research setting. The higher frequency of necrotizing enterocolitis in the control group was unexpected and, given the small number of events and the absence of adjustment, should be regarded as a chance or confounded observation rather than a true protective effect of intrauterine inflammation.

Overall, these findings are hypothesis-generating rather than confirmatory. They suggest that a single early postnatal measurement of serum neutrophil gelatinase-associated lipocalin, total oxidant status, or total antioxidant status is unlikely to provide a clinically useful classification of the type of intrauterine inflammatory exposure in extremely preterm infants. Adequately powered studies combining systematic placental histopathology with amniotic fluid or cord blood cytokine measurement, a more clearly defined comparison group, and serial biomarker sampling are needed to clarify the temporal behavior and potential clinical value of these markers.

## Figures and Tables

**Table 1:** Baseline characteristics of the study population.

Variable	Clinical Chorioamnionitis (*n* = 31)	Preterm Premature Rupture of Membranes (*n* = 33)	Control (*n* = 30)	*p*
Gestational age, weeks	27.0 [25.0–28.0]	27.0 [25.0–28.0]	26.0 [25.3–28.8]	0.922
Birth weight, g	1030 [815–1210]	980 [755–1225]	1020 [853–1108]	0.885
Cesarean delivery, n (%)	28 (90.3)	33 (100)	27 (90.0)	0.176
Maternal age, years	27.0 [23.5–31.0]	31.0 [28.0–34.0]	29.5 [26.0–34.8]	0.047
Antenatal corticosteroids, n (%)	17 (54.8)	19 (57.6)	12 (40.0)	0.332
Rupture-of-membranes-to-delivery latency, h	168 [24–240]	72 [27–160]	—	0.298

Data are median [interquartile range] or n (%). Comparisons among the three groups were made with the Kruskal–Wallis test or the Pearson chi-square test; the rupture-of-membranes-to-delivery latency was compared between the clinical chorioamnionitis and preterm premature rupture of membranes groups only, using the Mann–Whitney U test.

**Table 2:** Oxidant status, total antioxidant status, and serum neutrophil gelatinase-associated lipocalin among the study groups.

Biomarker	Clinical Chorioamnionitis (*n* = 31)	Preterm Premature Rupture of Membranes (*n* = 33)	Control (*n* = 30)	*p*
Serum neutrophil gelatinase-associated lipocalin, ng/mL	275.3 [146.1–525.6]	352.8 [275.3–2041.8]	343.1 [243.8–486.8]	0.031
Total antioxidant status, mmol Trolox equivalent/L	1.58 [1.39–1.98]	1.62 [1.39–2.01]	1.68 [1.38–1.95]	0.966
Total oxidant status, µmol hydrogen peroxide equivalent/L	22.9 [13.8–27.5]	18.0 [11.6–38.9]	18.8 [11.8–24.7]	0.525
Oxidative stress index, arbitrary units	1.11 [0.73–1.72]	1.18 [0.68–3.01]	1.09 [0.75–1.55]	0.729

Data are median [interquartile range]; Kruskal–Wallis test. Because the oxidative stress index is computed as a ratio for each infant, its group median is not equal to the ratio of the group medians of total oxidant status and total antioxidant status. Post hoc for neutrophil gelatinase-associated lipocalin: clinical chorioamnionitis versus preterm premature rupture of membranes, Bonferroni-adjusted *p* = 0.029.

**Table 3:** Multivariable linear regression model for log-transformed serum NGAL (*n* = 94).

Predictor	Adjusted β (95% CI) ^1^	*p* Value
Clinical chorioamnionitis vs. control	−0.285 (−0.85, 0.28)	0.322
PPROM vs. control	0.415 (−0.15, 0.98)	0.146
Gestational age (per week)	0.024 (−0.13, 0.18)	0.759
Birth weight (per 100 g)	−0.023 (−0.15, 0.10)	0.718
Maternal age (per year)	0.010 (−0.03, 0.05)	0.596
Antenatal corticosteroid (yes vs. no)	0.049 (−0.41, 0.51)	0.832
Clinical chorioamnionitis vs. PPROM	−0.700 (−1.27, −0.13)	0.017

Dependent variable: natural-logarithm-transformed serum NGAL; reference category for study group: control. ^1^ Analytic 95% confidence intervals; for the clinical chorioamnionitis-versus-PPROM contrast the bootstrap 95% CI was −1.29 to −0.14. Adjusted R^2^ = 0.02. NGAL, neutrophil gelatinase-associated lipocalin; PPROM, preterm premature rupture of membranes; CI, confidence interval.

**Table 4:** Neonatal outcomes by group.

Outcome	Clinical Chorioamnionitis (*n* = 31)	Preterm Premature Rupture of Membranes (*n* = 33)	Control (*n* = 30)	*p*
Bronchopulmonary dysplasia, n (%)	9 (29.0)	11 (33.3)	10 (33.3)	0.915
Necrotizing enterocolitis, n (%)	1 (3.2)	2 (6.1)	9 (30.0)	0.003
Patent ductus arteriosus, n (%)	3 (9.7)	7 (21.2)	6 (20.0)	0.410
Surfactant administration, n (%)	20 (64.5)	24 (72.7)	24 (80.0)	0.400
Positive blood culture, n (%)	2 (6.5)	2 (6.1)	2 (6.7)	0.995
Peripheral-smear toxic granulation, n (%)	5 (16.1)	10 (30.3)	2 (6.7)	0.049
Töllner sepsis score, median [IQR]	1.0 [1.0–1.0]	1.0 [1.0–2.0]	1.0 [1.0–2.0]	0.320
In-hospital mortality, n (%)	5 (16.1)	8 (24.2)	6 (20.0)	0.721

Data are median [interquartile range] or n (%). Necrotizing enterocolitis and blood culture comparisons used Fisher’s exact test; all other categorical variables were compared with the Pearson chi-square test, and the Töllner sepsis score with the Kruskal–Wallis test.

**Table 5:** In-hospital mortality by gestational-age band.

Gestational-Age Band	Infants, *n*	Deaths, *n*	Mortality
22–23 weeks	5	4	80.0%
24–25 weeks	23	10	43.5%
26–27 weeks	22	2	9.1%
28–29 weeks	31	3	9.7%
30–31 weeks	13	0	0%

The 22–23-week stratum comprised only five infants and should be interpreted with caution.

**Table 6:** Spearman correlation coefficients between serum biomarkers and selected clinical and laboratory variables (whole cohort); hematological, biochemical, and acute-phase parameters were measured at the 1st postnatal hour. Values are ρ with the uncorrected *p* value in parentheses.

Variable	*n*	Total Antioxidant Status	Total Oxidant Status	Oxidative Stress Index	Neutrophil Gelatinase- Associated Lipocalin
Total oxidant status	94	0.25 (0.017)	—	0.91 (<0.001)	0.00 (0.981)
Neutrophil gelatinase-associated lipocalin	94	−0.02 (0.838)	0.00 (0.981)	0.00 (0.997)	—
Oxidative stress index	94	−0.12 (0.253)	0.91 (<0.001)	—	0.00 (0.997)
C-reactive protein (1st hour)	94	0.14 (0.170)	−0.06 (0.577)	−0.14 (0.186)	−0.14 (0.164)
White blood cell count (1st hour)	93	0.05 (0.666)	−0.16 (0.133)	−0.22 (0.034)	0.01 (0.938)
Neutrophil count (1st hour)	93	0.21 (0.047)	0.03 (0.755)	−0.07 (0.517)	0.29 (0.005)
Platelet count (1st hour)	93	0.09 (0.417)	0.09 (0.399)	0.00 (0.990)	0.00 (0.970)
Procalcitonin (1st hour)	93	−0.01 (0.890)	0.19 (0.061)	0.25 (0.015)	0.03 (0.809)
Lactate dehydrogenase (1st hour)	93	−0.06 (0.579)	0.05 (0.658)	0.08 (0.418)	0.20 (0.051)
Lactate (1st hour)	51	0.14 (0.332)	0.07 (0.619)	0.07 (0.610)	0.03 (0.835)
Albumin (1st hour)	93	−0.05 (0.605)	0.19 (0.071)	0.18 (0.084)	0.03 (0.811)
pH (1st hour)	52	0.17 (0.242)	0.01 (0.942)	−0.07 (0.622)	0.24 (0.092)
Töllner sepsis score	94	−0.10 (0.331)	−0.17 (0.096)	−0.11 (0.306)	0.05 (0.613)
Gestational age	94	0.19 (0.064)	0.18 (0.075)	0.10 (0.314)	0.01 (0.951)
Birth weight	94	0.10 (0.330)	0.28 (0.007)	0.22 (0.033)	−0.07 (0.522)

Values are Spearman correlation coefficients (ρ) with the uncorrected *p* value in parentheses; n is the number of infants in whom both measurements were available, which varies because lactate and pH were not recorded in every infant. All clinical and laboratory parameters were measured at the 1st postnatal hour. These associations are exploratory: the Benjamini–Hochberg false-discovery-rate correction was applied across the 48 biomarker–parameter comparisons shown, and no coefficient reached statistical significance (smallest q = 0.17). The oxidative stress index is derived arithmetically from total oxidant status and total antioxidant status, so its correlations with those two measures are mathematically constrained and are shown for completeness only.

**Table 7:** Serum biomarker concentrations stratified by major neonatal outcomes (*n* = 94).

Outcome	Biomarker	With Event, Median [IQR]	Without Event, Median [IQR]	*p*	q (FDR)
Culture-positive sepsis (*n* = 6)	TAS	1.72 [1.30–2.16]	1.62 [1.39–1.96]	0.969	0.969
	TOS	17.8 [10.1–31.3]	19.7 [11.7–28.0]	0.693	0.963
	OSI	1.16 [0.66–1.67]	1.11 [0.73–1.76]	0.763	0.963
	NGAL	190 [128–348]	343 [238–720]	0.103	0.554
Necrotizing enterocolitis (*n* = 12)	TAS	1.55 [1.29–1.77]	1.64 [1.39–2.01]	0.316	0.843
	TOS	15.9 [9.9–32.8]	20.4 [12.4–27.7]	0.704	0.963
	OSI	0.90 [0.77–1.87]	1.13 [0.71–1.74]	0.816	0.963
	NGAL	282 [249–295]	371 [228–980]	0.104	0.554
Bronchopulmonary dysplasia (*n* = 30)	TAS	1.48 [1.30–1.82]	1.73 [1.42–2.01]	0.072	0.554
	TOS	19.2 [14.1–24.9]	20.4 [10.9–29.6]	0.862	0.963
	OSI	1.20 [0.88–1.73]	0.96 [0.70–1.81]	0.241	0.772
	NGAL	324 [220–723]	348 [233–667]	0.903	0.963
In-hospital mortality (*n* = 19)	TAS	1.62 [1.36–1.88]	1.66 [1.39–2.02]	0.489	0.963
	TOS	14.3 [11.2–24.4]	21.1 [12.6–30.5]	0.200	0.772
	OSI	0.82 [0.73–1.31]	1.16 [0.72–1.82]	0.371	0.848
	NGAL	350 [185–472]	340 [237–723]	0.572	0.963

Values are median [interquartile range]. *p* values are from the Mann–Whitney U test; q values are Benjamini–Hochberg false-discovery-rate-adjusted *p* values, and no comparison reached statistical significance after correction. TAS, total antioxidant status (mmol Trolox equivalent/L); TOS, total oxidant status (µmol H_2_O_2_ equivalent/L); OSI, oxidative stress index (arbitrary units); NGAL, neutrophil gelatinase-associated lipocalin (ng/mL).

**Table 8:** Spearman correlation coefficients (ρ) between the first-hour serum biomarkers and laboratory parameters measured at the 24th postnatal hour. Values are ρ with the uncorrected *p* value in parentheses. No coefficient reached statistical significance before or after Benjamini–Hochberg false-discovery-rate correction across the 20 comparisons (all q ≥ 0.539). These analyses are exploratory.

Parameter at the 24th Postnatal Hour	*n*	Total Antioxidant Status	Total Oxidant Status	Oxidative Stress Index	Neutrophil Gelatinase- Associated Lipocalin
C-reactive protein	89	−0.02 (0.872)	−0.18 (0.092)	−0.17 (0.108)	0.06 (0.551)
White blood cell count	90	−0.06 (0.545)	−0.07 (0.540)	−0.10 (0.349)	0.17 (0.108)
Neutrophil count	90	0.06 (0.589)	0.03 (0.783)	−0.04 (0.739)	0.19 (0.066)
Platelet count	90	0.05 (0.635)	0.14 (0.199)	0.07 (0.507)	−0.01 (0.889)
Procalcitonin	73	−0.05 (0.678)	0.05 (0.696)	0.11 (0.337)	−0.06 (0.598)

Data are Spearman correlation coefficients (uncorrected *p* values). Procalcitonin was not measured routinely at all sites at 24 h, hence the smaller number of observations.

## Data Availability

The data presented in this study are available from the corresponding author upon reasonable request. The data are not publicly available because they contain information that could compromise the privacy of the participants and are subject to institutional and ethical restrictions.
